# The effect of smiling on the perceived age of male and female faces across the lifespan

**DOI:** 10.1038/s41598-021-02380-2

**Published:** 2021-11-26

**Authors:** Tzvi Ganel, Melvyn A. Goodale

**Affiliations:** 1 Psychology Department, Ben-Gurion University of the Negev, 8410500 Beer-Sheva, Israel; 2 The Brain and Mind Institute, The University of Western Ontario, London, ON N6A 5B7 Canada

**Keywords:** Human behaviour, Perception

## Abstract

Previous research has shown an unintuitive effect of facial expression on perceived age: smiling faces are perceived as older compared to neutral faces of the same people. The aging effect of smiling (AES), which is thought to result from the presence of smile-related wrinkles around the eyes, contradicts the common belief that smiling faces should be perceived as younger, not older. Previous research, however, has focused on faces of young adults, where the absence of inherent, age-related wrinkles and other age signs is offset by the weight of the smile-related wrinkles. In a series of experiments, we tested whether the AES extends to male and female faces in older age groups. We replicated the AES in young adults (20–39) and showed that it disappeared in older adults (60–79) of both genders. For photos of middle-aged adults (40–59), however, AES was found only for male, but not for female faces, who showed fewer and less prominent smile-related wrinkles. The results suggest that a person’s apparent age is perceived in a holistic manner in which age-related cues in the region of the eyes are weighted against age cues in other regions of the face.

## Introduction

Faces are considered as multidimensional stimuli that convey various aspects of social information. Such dimensions include a person’s identity, gender, age, race, expression, trustworthiness, among other dimensions that constitute a person’s social world^1–3^. Notably, previous research highlighted the interactive manner in which these different dimensions are perceived. For example, facial expression cannot be perceived independently of the person’s identity^4,5^, which argues for shared functional and neural mechanisms of processing these dimensions^6,7^. Similar interactions were observed between the processing of identity, expression, and gender^8–10^, identity and age^11^, and expression and trustworthiness^12^. The interactive manner in which different facial dimensions are perceived is also reflected in the unique, holistic processing style of faces compared to other visual stimuli^13^. Therefore, just as the processing of different facial features interact to create a coherent holistic percept, different facial aspects are processed in an interactive manner when perceivers evaluate the expression, the identity, or the age of a face.

Here, we focused on the processing of a person’s age and its relation to changes in facial appearance related to the formation of expression. Among the many dimensions that perceivers readily extract when viewing unfamiliar (and familiar) faces, age is considered as primary^14^. Accurate identification of a person’s age is crucial for understanding social roles and determining the nature of social interaction^15^. Unfortunately, although faces provide rich sources of cues about the real age of a person (e.g., wrinkles, facial outline, skin pigmentation, and hair style and colour) people are often inaccurate in extracting real age information from viewing faces^15,16^. Reasons can be attributed to two main sources; first, different people age differently, as a function of both genetic and environmental factors^17,18^. Second, and more relevant to the present investigation, perceivers do not always efficiently extract available age cues from faces, and sometimes use irrelevant visual information as informative for age.

One example of people’s inefficiency in age perception is the directional bias induced by smiling. In previous studies from our lab, we showed that smiling faces are perceived as older (between one and two years) compared to faces of the same people when they display a neutral expression. This aging effect of smiling (AES) is thought to be the result of the formation of smile-related wrinkles around the region of the eyes^19^. Indeed, the AES is diminished or significantly reduced when wrinkling information is graphically removed or when only the bottom part of the face is presented. In contrast, when images of the top part of the face are presented for age evaluation, the AES becomes even larger^19^.

Perhaps even more interesting is the fact that the AES stands in direct contrast to the common belief that smiling makes one look younger. The reasons for this mistaken belief are not clear. Smiling faces have been shown to be associated with positive values such as attractiveness^20^. Similarly, a youthful face is associated with a positive evaluation^15^. Perhaps it is not surprising, therefore, that people commonly believe that smiling is associated with perceived youth. Indeed, when we asked participants whether smiling faces are perceived as younger or older, most participants expressed the belief that smiling faces are perceived as younger. Remarkably, during the same experimental session, direct perceptual estimations of age showed that smiling faces were perceived as older than neutral faces, but at the same time, retrospective evaluations of the age of the same set of faces went in exactly the opposite direction^21^. In short, in contrast to common belief, smiling faces are perceived as older than neutral faces.

The AES has been recently replicated and extended to faces of other races^22^. In particular, significant AES were found for age evaluations of Swedish and Japanese faces by both Swedish and Japanese participants. In addition, in contrast to participants’ actual judgments of the age of smiling and neutral faces, their retrospective evaluations of age again followed the incorrect common belief that smiling faces should be perceived as younger^22^.

Previous literature that looked at the effect of smiling on age perception focused on the perception of the faces of young adults, 20 to 40 years of age. Faces of young adults typically have a smaller number of inherent, age-related wrinkles as well as fewer signs of age, such as hair colour and skin pigmentation, compared to older adults. Therefore, the formation of smile-related wrinkles can easily offset other cues to age in the faces of young adults, triggering the AES. But what happens when we judge the age of the smiling faces of older adults? The faces of older adults contain a substantial number of age-related wrinkles and other age cues compared to faces of younger adults. Given that faces are perceived in an holistic manner, it is possible that the temporary formation of smile-related wrinkles, when judged in the context of the entire face, would have smaller effect on judgments of the age of older adults. The main purpose of the current study was to test the generality of the aging effect of smiling across different age groups. To do this, we ran a series of experiments in which participants judged the age of smiling and neutral faces of young, middle-aged, and old adults. To test for possible effects of gender, we compared the AES of male and female faces of different age groups.

## Experiments 1a and 1b

In Experiments 1a and 1b, participants made age evaluations of a series of photos of smiling and neutral faces of three different age groups. Photos of the entire face were presented in Experiment 1a and photos of the eye region of the same faces were presented in Experiment 1b. This design allowed us to measure the AES across different age groups as well as isolating the role of smile-related wrinkles in the region of the eyes in mediating this effect.

Sample sizes were determined based on our previous experiments. The effect-size of AES in Ganel & Goodale (2018) was partial eta squared = 0.39 for young adult faces. Given that no previous studies have examined this effect across different age groups, when determining the required sample size for the present study, we focused on the AES. An a priori power analysis was conducted using G*Power. The required sample size for achieving 80% power to detect a much smaller effect size (partial eta squared = 0.05), with a correlation of 0.8 between the conditions (based on the data of Ganel & Goodale, 2018), was 17. We therefore aimed at a minimum number of 17 participants in each experiment. The sample sizes in experiments 1a, 1b, and 2 were 30, 24, and 27, respectively. A smaller sample size of 21 was used in Experiment 3, due to relative difficulty in recruiting middle-aged adult participants. Still, all sample sizes exceed the initial power requirement.

### Method

#### Participants

Thirty Ben Gurion University of the Negev students (12 males, mean age = 23.4 years, SD = 1.63 years) participated in Experiment 1a and 24 different students (7 males, mean age = 24.46 years, SD = 1.42) participated in Experiment 1b. All participants had normal or corrected-to-normal vision and received course credit for their participation. The experimental protocol in Experiment 1 was approved by the ethics committee of the Department of Psychology in Ben-Gurion University of the Negev. The study adhered to the ethical standards of the Declaration of Helsinki. All participants signed an informed consent form prior to their participation in the experiment. The manuscript contains no information or images that could lead to identification of a study participant.

#### Design and materials

**Figure 1:**
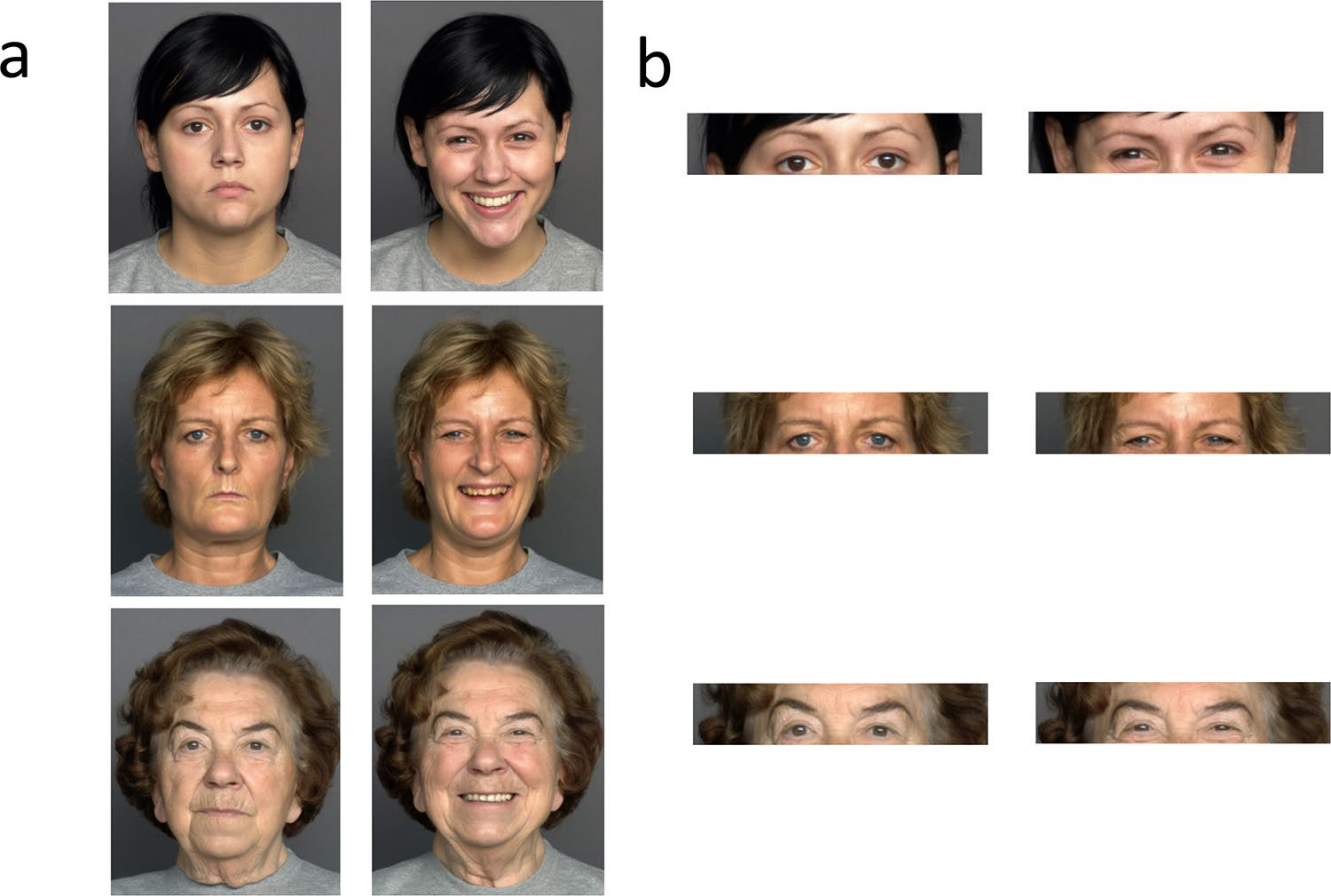
Sample stimuli used in Experiment 1a and Experiment 1b. (**a**) Full neutral and smiling faces of young adults, middle-aged adults, and old adults presented in Experiment 1a. (**b**) Cropped photos of the eye region of the same faces presented in Experiment 1b. Adapted from Ebner, N. C., Riediger, M., & Lindenberger, U. (2010). FACES—A database of facial expressions in young, middle-aged, and older women and men: Development and validation. Behavior Research Methods, 42, 351–362. 10.3758/BRM.42.1.351, all rights reserved.

Photos of 120 women and 120 men, all with neutral and smiling expressions, were taken from different face databases that included information on the actual age of the person at the time the photograph was taken. The faces were taken from the FACES database (161 faces)^16^, the PAL face database (66 faces)^23^, and from a set of faces photographed by members of the Ganel lab (13 faces). The photos were equally divided to 3 age groups: young adults (20–39 years), middle-aged adults (40–59), and old adults (60–79 years). Faces of 40 female and 40 male faces were included in each age group. Due to constraints inherent to the sets, there were small variations within the average age and within the age range in each age group. The average age of the young adult group was 24.94 years old (24.93 for female faces, 24.95 for male faces) with a range of 20–39 years. The average age of the middle-aged adult group was 49.1 years old (49.83 for females, 48.38 for males) with a range of 41–58 years. The average age of the old adult group was 71.39 years old (71.3 for females, 71.48 for males) with a range of 61–79 years. Stimuli were cropped to the dimension of about 18 X 13 cm in size, depending on the database, and presented on a 19" screen using E-Prime software. The photos of the same faces were used in Experiment 1b, but were cropped to include only the eye region of the face (Fig. 1).

#### Experimental procedure

The procedures used in Experiments 1a and 1b were similar, except that photos of full faces were presented in Experiment 1a while photos of only the eye region were presented in Experiment 1b. The experimental design was adapted from previous studies in our lab^19,21^. The stimulus set was divided into two equal sets of 120 photos (sets A and B, each set included the faces of 20 females and 20 males in each age group). For half of the participants, the faces in set A were smiling and those in set B displayed a neutral expression. For the other half of the participants, set B was smiling and set A had a neutral expression. Participants were presented with one face on each trial, and were asked to evaluate the age of the face as accurately as possible. The faces from sets A and B and from the different age groups were presented in a random order. Each face was presented on the screen until a response was made. Participants typed their response in years, which appeared below the target photo, and then pressed the "Return" key to indicate that they had completed their response, after which they proceeded to the next trial. Responses that were not 2-digit numbers were excluded from the analysis (less than 1% of the total responses).

### Results and discussion

#### Experiment 1a: full faces

**Figure 2:**
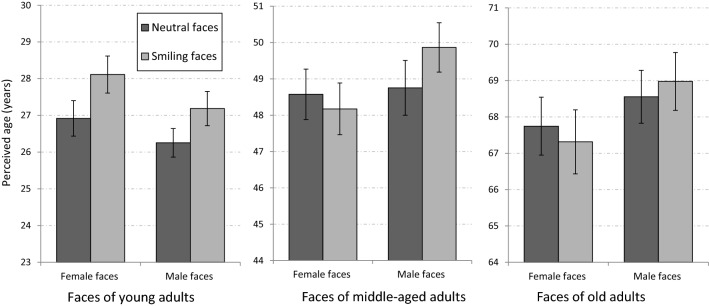
The aging effect of smiling (AES) in different age groups of faces in Experiment 1a (full faces). AES was found for male and for female faces in young adults, but only for male faces in middle-aged adults. No AES was found for faces of old adults. Error bars represent standard errors of the mean.

The mean perceived ages of neutral and smiling faces in the different age groups are presented in Fig. 2. As can be seen in the figure, an aging effect of smiling (AES) was found for both female and male faces of young adults. In sharp contrast, there was no evidence of AES for the faces of older adults. For faces of middle-aged adults, AES was found in male, but not in female faces.

A mixed ANOVA design with the gender of the photographed person, his/her expression (smiling vs. neutral) and age group as the within-subject independent variables and with the participant’s gender as a between-subjects independent variable was used to analyse the data, with perceived age (years) serving as the dependent variable. The participant’s gender did not have a significant effect on any AES in this or in any of the remaining experiments, and will not be discussed further in the context of the AES.

Not surprisingly, a main effect was found for age group [F(2,56) = 3826.8, p < 0.001, η_p_^2^ = 0.99]. A main effect was also found for expression [F(1,28) = 6.35, p = 0.018, η_p_^2^ = 0.18], indicating that overall, smiling faces were perceived as older than neutral faces. The main effect of gender (of the photographed person) was not significant [F(1,28) = 1.77, p = 0.19, η_p_^2^ = 0.06]. A significant interaction between expression and gender [F(1,28) = 8.73, p = 0.006, η_p_^2^ = 0.24] indicated that overall, the AES was larger for male compared to female faces. However, this interaction was modulated by interactions with other variables and, as described below, was explored using specific comparisons. A significant interaction was also found between age group and expression [F(2,56) = 3.54, p = 0.036, η_p_^2^ = 0.11], indicating different effects of smiling on perceived age in different age groups. In addition, an interaction was found between age group and gender [F(2,56) = 9.46, p < 0.001, η_p_^2^ = 0.25], indicating that the age differences between male and female faces were affected by age group. The interaction between participant’s gender and between age group was also significant [F(2,56) = 3.29, p = 0.045, η_p_^2^ = 0.11], indicating that the differences between age evaluation of female and male participants were moderated by the age group of the photographed face. Finally, a 3-way interaction between age group, gender, and expression [F(2,56) = 4.29 p = 0.019, η_p_^2^ = 0.13], indicated that the interaction between gender and expression was expressed differently in the different age groups. To better understand the pattern of the results and interactions, we performed specific comparisons of age evaluations of smiling and neutral female and male faces within each age group.

For faces of young adults, female faces were perceived as older than male faces [F(1,28) = 11.6, p = 0.002]. More importantly, an aging effect of smiling (AES) was found for both female [F(1,28) = 25.22, p < 0.001], and male faces [F(1,28) = 11.48, p = 0.002]. This result replicates and extends previous findings of AES in faces of young adults^19,21^. The interaction between gender and expression was not significant [F(1,28) < 1].

For faces of middle-aged adults, female faces were perceived as younger than male faces [F(1,28) = 5.15, p < 0.031]. More importantly, an aging effect of smiling (AES) was found for male faces [F(1,28) = 4.79 p <  = 0.037], but there was no effect of smiling for female faces [F(1,28) < 1]. The different patterns of AES for male and female faces were indicated by a significant interaction between gender and expression [F(1,28) = 9.96 p = 0.004]. This pattern of results was unexpected and shows that the AES in middle-aged adults depends on the gender of face. We will discuss possible sources for this effect later in the discussion section.

For faces of old adults, and similarly to faces of middle-aged adults, female faces were perceived as younger than male faces [F(1,28) = 4.62, p = 0.04]. As predicted, AES was not found for faces in this age group, neither for female faces [F(1,28) = 1.94, p = 0.17], nor for male faces [F(1,28) = 1, p = 0.32]. The interaction between gender and expression was not significant [F(1,28) = 3.82, p = 0.06].

We computed accuracy scores for age evaluations for full faces in Experiment 1a (and in Experiment 3). Accuracy was computed by calculating the average absolute difference between the perceived and real age in each combination of age group, gender, and expression. Accuracy scores for the different conditions are presented in Table 1. As can be seen in the table, accuracy was overall higher for young adult faces compared to the other age groups and was also higher for neutral compared to smiling faces across all age groups. This pattern of results is similar to the one reported by Voelkle et al.^15^.

**Table 1:** Mean accuracy (absolute errors in years) of age evaluations of full faces in the different age groups in experiments 1a and 3 (standard deviations in brackets).

Age group (of faces)		Young adults		Middle-aged adults		Old adults	
Gender (of faces)		Female	Male	Female	Male	Female	Male
Experiment 1a (participants: young adults)	Neutral faces	4.46 (1.3)	4.42 (0.9)	6.32 (1.5)	6.44 (1.8)	7.34 (2.5)	6.65 (2)
	Smiling faces	5.03 (1.6)	5.32 (1.3)	6.82 (1.8)	6.6 (1.5)	7.89 (2.6)	6.77 (2)
Experiment 3 (participants: middle-aged adults)	Neutral faces	5.31 (2.1)	5.55 (1.9)	6.39 (1.1)	7.3 (2.3)	6.29 (1.5)	5.65 (1.3)
	Smiling faces	6.22 (2.1)	6.82 (2.3)	6.55 (1.5)	7.73 (2.4)	6.83 (1.6)	5.73 (1.4)

## Experiment 1b: eye region only

**Figure 3:**
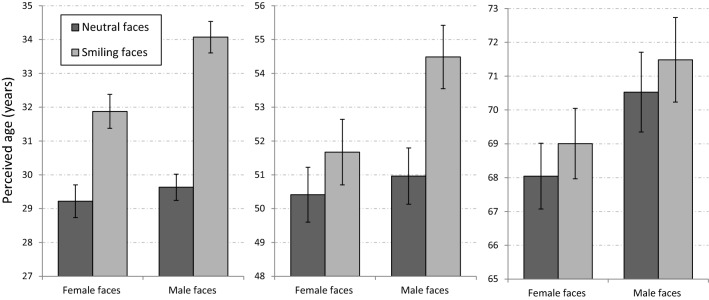
The AES for the eye region of faces in different age groups (Experiment 1b). Smiling faces were perceived as older than neutral faces for both genders and in all age groups. Error bars represent standard errors of the mean.

Mean perceived ages of neutral and smiling faces in the different age groups are presented in Fig. 3. As can be seen in the figure, the pattern of results for the eye region of the faces in Experiment 1b was different from the pattern of results observed for the entire faces in Experiment 1a. In particular, an aging effect of smiling (AES) was evident for the eye region in all age groups for both female and male faces.

A mixed ANOVA with gender, expression, and age group as the within-subject independent variables and with the participant’s gender as a between-subjects independent variable revealed main effects of age group [F(2,44) = 831.58, p < 0.001, η_p_^2^ = 0.97] and of gender [F(1,22) = 28.82, p < 0.001, η_p_^2^ = 0.57], indicating that overall, the eye region of female faces was perceived as younger than that of male faces across all age groups. More importantly, there was a main effect of expression [F(1,22) = 69.86, p < 0.001, η_p_^2^ = 0.76], indicating that smiling faces were perceived as older than neutral faces. A significant interaction between expression and gender [F(1,22) = 25.17, p < 0.001, η_p_^2^ = 0.53] indicated that overall, as in Experiment 1a, the AES for the eye region was larger for male compared to female faces. As in Experiment 1a, an interaction was found between age group and expression [F(2,44) = 7.72, p = 0.001, η_p_^2^ = 0.26], due to the larger AES in young adults and in middle-aged adults compared to old adults. The interactions between age group and gender [F(2,44) = 1.25, p = 0.29, η_p_^2^ = 0.05], and between age group, gender, and expression [F(2,44) = 2.81, p = 0.071, η_p_^2^ = 0.11] were not significant.

Unlike in Experiment 1a, the effect of smiling on perceived age was robust in the different age groups. Specific comparisons showed significant AES in the young age group for both genders (F(1,22) = 52.85, p < 0.001 for female faces; F(1,22) = 59.3, p < 0.001 for male faces). A similar trend was found in the old adult group (F(1,22) = 3.86, p = 0.06 for female faces; F(1,22) = 6.04, p = 0.022, for male faces). Interestingly, the pattern of results for the eye region of middle-aged adults echoed that of the full faces of middle aged-adults in Experiment 1a. In particular, a significant AES was found for only for male faces [F(1,22) = 16.08, p < 0.001], but not for female faces [F(1,22) = 1.32, p = 0.26]. A significant interaction between gender and expression in the middle-aged group [F(1,22) = 12.31, p = 0.001] indicated differences in the AES between female and male faces within this age group.

### Analysis across Experiments 1a and 1b

To test the idea even further that the AES is enhanced when participants view only the eye region of the face, we performed an additional between-subject analysis across Experiments 1a and 1b. A mixed ANOVA was performed on the data from the two experiments with gender, expression, and age group as the within-subject independent variables and with the participant’s gender and presentation format (full faces, Experiment 1a vs. eye region, Experiment 1b) as between-subject independent variables. Our focus was on the effects of expression and presentation format on average age perception, and for sake of brevity, we will not go into details in describing other effects and high-level interactions, as well as nonsignificant effects in this analysis.

The analysis revealed main effects of age group [F(2,100) = 3279.59, p < 0.001, η_p_^2^ = 0.98], gender [F(1,50) = 25.11, p < 0.001, η_p_^2^ = 0.33], and expression [F(1,50) = 76.73, p < 0.001, η_p_^2^ = 0.61]. There was also a significant main effect of presentation format [F(1,50) = 10.34, p = 0.002, η_p_^2^ = 0.17], with full faces perceived as younger than photos that include only the eye region of the face. More importantly, a significant interaction between presentation format and expression [F(1,50) = 76.73, p < 0.001, η_p_^2^ = 0.61] indicated that the AES was overall larger in Experiment 1b in which only the eye region was presented, compared to the presentation of the entire face in Experiment 1a. As in Experiments 1a and 1b, there was a significant interaction between age group and expression [F(2,100) = 12.48, p < 0.001, η_p_^2^ = 0.19], indicating larger AESs in the young adults and in middle-aged adults compared to old adults. The results of this analysis reinforce the notion that the AES is triggered by wrinkles around the region of the eyes.

The results of Experiment 1 replicate and extend previous evidence on the relation between smiling and perceived age. Smiling faces of young adults were again perceived as older. This effect was diminished for the faces of old adults, for whom we predicted that the influence of smile-related wrinkling would be diminished by other substantial age cues. Most interesting are the results for the middle-aged faces. Here, we found an unexpected modulation of the AES by the gender of the face; in particular, smiling male faces were perceived as older than smiling neutral faces. For female faces, however, there was no effect of smiling on the perceived age. One reason for this interaction between gender and facial expression was provided by the results on Experiment 1b, in which only the eye region of the faces was presented for view. In this experiment, AES was found for middle-aged male faces, but not for middle-aged female faces. It is possible therefore that the absence of a smiling effect in female faces is related to the weaker smile-related wrinkling effect in middle-aged females, which is not sufficient to trigger AES when the faces are presented as wholes.

Another possible account for the interaction between AES and gender in middle-aged adults could be related to the use of makeup. In particular, it is possible that for middle-aged adults, the use of makeup in female faces could have attenuated the AES. To rule out this possibility, we examined the effect of AES in the different databases we used in Experiment 1a. The database we used contained 240 faces overall, derived from three different sources: The FACES database (161 faces), faces photographed in Ganel’s lab (13 faces), and the PAL face database (66 faces). Models photographed for the two first databases were asked not to wear makeup (174 faces out of 240 overall). There was no such explicit requirement mentioned in the description of the PAL database. However, visual inspection of the faces in that set does not indicate a noticeable use of makeup. Still, to rule out the idea that the interaction between gender and AES in was affected by the possible use of makeup in female faces, we reanalyzed the data of Experiment 1 only for faces taken from the FACES and from Ganel’s lab databases, for which makeup was not allowed. The pattern of results was remarkably similar to the one obtained in the main analysis. Of particular interest, for faces of middle-aged adults, AES was found for male faces [F(1,29) = 10.71, p = 0.003], but not for female faces [F(1,29) = 1.86, p = 0.18]. The interaction between the gender of the face and between expression was again significant, [F(1,29) = 22.04, p < 0.001].

We will discuss other possible sources for this gender-related interaction later in the paper, but first, we report the results of an experiment in which we examined whether or not there was a more direct relation between smiling and the perception of wrinkles of middle-aged faces. To do this, participants were presented with only the eye region of middle-aged female and male faces, but instead of asking them to make an age evaluation, we asked them to rate the prominence of wrinkles in each face. To the extent that the modulation of the AES by gender is modulated by the perception of smile-related wrinkles, we predicted that smiling, compared to neutral faces, would result in more prominent wrinkles in male compared to female faces.

## Experiment 2

### Participants

Twenty-seven students from the Psychology department of Ben Gurion University of the Negev (8 males, mean age = 27 years, SD = 3.45 years) participated in Experiment 2. Participants were graduate and undergraduate students from Ganel's lab and other labs in the Psychology department, who volunteered to participate in the experiment. Participants were not informed about the purpose the experiment. The experiment was run online using Qualtrics. The experimental protocol in Experiment 2 was approved by the ethics committee of the Department of Psychology in Ben-Gurion University of the Negev. The study adhered to the ethical standards of the Declaration of Helsinki. All participants signed an informed consent form prior to their participation in the experiment.

### Procedure and design

The same photos of the eye region of the middle-aged faces used in Experiment 1b (40 women and 40 men) with neutral and with smiling expressions, were presented in the experiment. As was the case in Experiment 1, the stimuli were divided into two sets of neutral and smiling faces, which were counterbalanced between participants (10 participants were presented with set A showing a neutral expression and with set B showing a smiling expression, 17 participants were presented with set A smiling and set B with a neutral expression). As was the case in Experiment 1b, participants were presented with one face on each trial, but now they were asked to directly evaluate the prominence of wrinkles in each photo using a Likert 1–5 scale (1-not prominent, 5-very prominent). Faces were presented in a random fashion. Each face was presented on the screen until a response was made. Participants indicated their response using a computer mouse on a 1–5 scale, which appeared below the target photo, and then pressed the "Next" option to indicate that they had completed their response after which they proceeded to the next trial.

### Results and discussion

**Figure 4:**
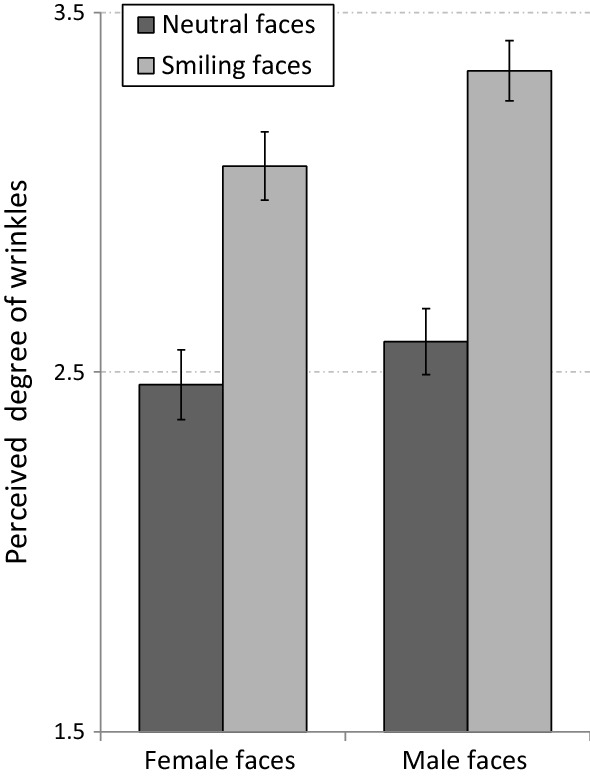
Mean rating of the prominence of wrinkles in the eye region of middle-aged faces. Smiling faces were perceived as containing a greater degree of wrinkling than neutral faces. In addition, male faces were perceived as containing more wrinkles than female faces, an effect which was found mostly for smiling faces. Error bars represent standard errors of the mean.

The perceived degree of wrinkling in the eye region of middle-aged female and male faces is presented in Fig. 4. As can be seen in the figure, the degree of wrinkling around the eyes was rated as larger for male faces, especially when they were smiling.

A mixed ANOVA design with gender (of the face) and expression (smiling vs. neutral) as the within-subject independent variables and with the participant’s gender as a between-subjects independent variable was used to analyse the data. The perceived degree of wrinkling served as the dependent variable. The analysis revealed main effects for the expression [F(1,25) = 131.89, p < 0.001, η_p_^2^ = 0.84] and for the gender of the faces [F(1,25) = 10.75, p = 0.003, η_p_^2^ = 0.3]. These results indicate that the eye region of smiling faces was perceived as containing more prominent wrinkles than neutral faces and that male faces were perceived as containing more prominent wrinkles than female faces. More importantly, these main effects were qualified by a significant interaction between gender and expression [F(1,25) = 5.81, p <  = 0.024, η_p_^2^ = 0.19]. Specific comparisons showed that this interaction resulted from a disproportionately larger difference between the perceived degree of wrinkling in male and female faces for smiling faces. For neutral faces, the perceived degree of wrinkling did not statistically differ between male and female faces [F(1,25) = 2.25, p = 0.15]. For smiling faces, however, male faces were rated as containing a significantly more wrinkling than female faces [F(1,25) = 13.89, p > 0.001].

The results of Experiment 2 reinforce our hypothesis that in middle-aged adults, the prominence of smile-related wrinkles is disproportionately smaller in female compared to male faces. These results converge with the pattern of results obtained in Experiment 1, in which middle-aged smiling females were not perceived to be older than the same faces with neutral expressions. The absence of AES for female compared to male faces was evident in Experiment 1 both for full faces, but also for the eye region of the same faces. Again, the results show that unlike middle-aged male faces, middle-aged female faces do not show a notable increase in wrinkles during smiling. Possible reasons for this effect will be discussed later.

One potential concern related to the results of Experiments 1 and 2 is the possibility that the different expression of the AES across the different age groups may be related to the age group of our participants. In previous experiments, the participants were young adults. It could be the case, therefore, that the age of the participants interacted with the age of the presented faces and contributed to the different pattern of AES across the age groups. This concern mainly involves the pattern of results for middle-aged adults, for which there was an intriguing interaction between the gender of the face and the effect of smiling on age judgments. Could this interaction result from the fact that the participants in Experiment 1 were young adults? To resolve this issue, we ran an additional experiment with the same design as the one used in Experiment 1a, with middle-aged participants. This allowed us to test if the findings of Experiment 1a could be replicated in this group.

## Experiment 3

### Participants

Twenty-one middle-aged participants (9 males, age range between 41–60, mean age = 53.5 years, SD = 5.95 years) volunteered to participate in the experiment. The participants were the parents and relatives of students in the Ganel lab, and were not informed about the purpose of the experiment prior to their participation. All participants had normal or corrected-to-normal vision. Ethics were approved by the local ethics committee and participants signed an informed consent form prior to their participation in the experiments.

### Procedure and design

The design and procedure of the experiment were identical to those used in Experiment 1a. Only full faces were presented. The only exception was that the experiment was run on a laptop in the participants’ houses rather than in the lab. The experimental protocol in Experiment 3 was approved by the ethics committee of the Department of Psychology in Ben-Gurion University of the Negev. The study adhered to the ethical standards of the Declaration of Helsinki.

### Results and discussion

**Figure 5:**
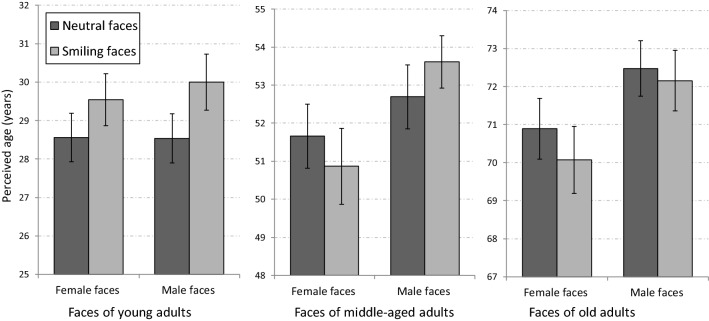
The aging effect of smiling (AES) in different age groups of faces in Experiment 3 (full faces). Participants were middle-aged adults. Note, that as in Experiment 1a, for faces of middle-aged adults, AES was found only for male faces, but not for female faces. No AES was found for faces of old adults. Error bars represent standard errors of the mean.

The mean perceived ages of neutral and smiling faces in the different age groups are presented in Fig. 5. As can be seen in the figure, the overall pattern of results resembles the pattern of results obtained in Experiment 1a for young adult participants. Again, an aging effect of smiling (AES) was found for the faces of young adults both for female and male faces, with no AES for the faces of older adults. As was the case in Experiment 1a, the AES in the middle-aged group was found only for male not female faces.

A mixed ANOVA with gender (of the face), expression, and age group as within-subject independent variables and with the participant’s gender as a between-subjects independent variable was performed on the data. The analysis revealed main effects for age group [F(2,38) = 1695.1, p < 0.001, η_p_^2^ = 0.98] and for gender of the face [F(1,19) = 15.6, p < 0.001, η_p_^2^ = 0.45], indicating that overall, female faces were perceived as younger than male faces. The main effect of expression was not significant [F(1,19) = 1.03, p = 0.32, η_p_^2^ = 0.05]. As in Experiment 1a, a significant interaction between age group and expression [F(2,38) = 10.49, p < 0.001, η_p_^2^ = 0.36] indicated different effects of smiling in the different age groups. As in Experiment 1a, a significant interaction was found between age group and gender [F(2,38) = 4.81, p = 0.014, η_p_^2^ = 0.2] indicating that the differences between age evaluations of female and male participants were modulated by the age of the face. In addition, a significant interaction between the gender and expression of the face [F(1,19) = 5.71, p = 0.027, η_p_^2^ = 0.23], indicated the overall, the AES was larger for male than for female faces. The 3-way interaction between age group, gender and expression was not significant [F(2,38) = 1.84, p = 0.17, η_p_^2^ = 0.09].

Specific comparisons showed a significant AES in the young age groups for both genders (F(1,19) = 9.76, p = 0.006 for female faces; F(1,19) = 12.53, p = 0.002 for male faces). Again, no AES was found in the old adult group (F(1,19) = 2.73, p = 0.11 for female faces; F(1,19) = 1.1, p = 0.31, for male faces). As in Experiment 1a, a significant AES was found for male faces in the middle-aged group [F(1,19) = 5.98, p = 0.025], but not for female faces [F(1,19) = 2.82, p = 0.11].

Accuracy scores for the different conditions are presented in Table 1. As can be seen in the table, there was no indication for an “own-age bias” (middle-aged adults participants did not evaluate the age of middle-aged adults more accurately than young adult participants (Experiment 1a)). Instead, the data show that middle-aged adult participants were less accurate overall in age evaluations compared to young adult participants for young and middle-aged adults faces. We note that a similar pattern of results was reported by Voelkle et al.^15^.

The results of Experiment 3 replicate and extend the results of Experiment 1, but in a different population of participants. In Experiment 3, middle-aged participants showed a pattern of results which highly resembles the pattern of results obtained for young adult participants in previous experiments. Again, AES was found for both male and female faces of young adults. Again, AES was absent for male and female faces of old adults. Most importantly, for middle-aged faces, AES was found for male, but not for female faces.

## General discussion

The results of our experiments replicate those of earlier studies from our lab and others showing that when people smile they are perceived as being one or two years older than when they maintain a neutral expression^19,21,22^. More importantly, however, the current experiments make it clear that this effect is modulated by a person’s real age. The faces of young people in their 20 s and 30 s in our experiments showed a strong and reliable effect of smiling on perceived age whereas people over the age of 60 showed little or no effect. The story with respect to middle-aged people, however, is complicated by an unexpected gender difference. Although middle-aged males appeared to be older when they smiled, there was no effect of smiling on the perceived age of middle-aged females. Moreover, this gender-based difference in the effect of smiling was not a function of the age of the observers: both young and middle-aged participants showed the same pattern of results. As we discuss below, the striking difference in the effects of smiling on the perceived age of middle-aged men and women, as well as the overall effect of smiling on perceived age across the entire range of ages that we tested, are due to differences in the wrinkling around the eyes as a function of smiling, age, and, in the case of middle-aged faces, gender. It is worth noting that all the faces presented in our study were Caucasian. Although the age-increasing effect of smiling has been observed in young Japanese faces^22^, it is not yet known whether middle-aged faces of other races would show the gender difference we observed.

When photos of only the region around the eyes were presented to observers (Experiment 1b), the effect of smiling was not only still present but was also enhanced. This suggests that the wrinkling around the eyes (i.e., crow’s feet), which increases when people smile, is a major driver of the age-increasing effect of smiling. Moreover, a comparison between the age ratings of full face (Experiment 1a) and eye-region only photos (Experiment 1b) revealed that full faces were perceived to be younger than eye-region only photos, whether or not they were smiling. In other words, when no other facial cues to age were available, judgements of age had to be based entirely on the wrinkling around the eyes, and thus eye-region only photos were perceived as older than full face photos.

But even with the eye-only photos, the gender difference that we observed for middle-aged full faces, namely that smiling makes males but not females look older, was preserved. Could this be due to differences in the prominence of wrinkles in the eye region of middle-aged male and female faces? When we asked participants to rate the prominence of eye wrinkles in photos showing only the eye region of middle-aged faces, they definitely rated smiling faces as having more wrinkles than faces with a neutral expression. This finding, in itself, provides strong confirmation of the contribution of eye wrinkles to the perception of a person’s age. But critically, observers also rated the degree of wrinkling around the eyes as greater for male faces than female faces, and this difference was more prominent in smiling faces.

Taken together, these results suggest that smiling has a smaller effect on the perceived age of middle-aged females than males – and this difference is driven by the fact that middle-aged females have fewer wrinkles around the eyes than males, due to smiling. This result is consistent with the fact that there are conspicuous anatomical and physiological differences in the skin of men and women (for review^24^). Importantly, the faces of middle-aged men show larger areas of wrinkled skin than middle-aged women, associated with life style differences, such as smoking, outdoor working history, and alcohol consumption^25^. With respect to crow’s feet, which are most prominent when people smile, middle-aged women are more likely than middle-aged men to use skin products^26^ and even Botulinum toxin injections^27^ to deal with these wrinkles and other facial cues to aging. Thus, for all these reasons, it is perhaps not surprising that the effect of smiling on perceived age in middle-aged women is not nearly as strong as it is in middle-aged men. In old age, however, the difference in the effects of smiling on perceived age between men and women disappears, presumably because other cues to age, including wrinkled skin elsewhere on the face and neck, are prominent in both genders.
